# Coupling proteomics and metabolomics for the unsupervised identification of protein–metabolite interactions in *Chaetomium thermophilum*

**DOI:** 10.1371/journal.pone.0254429

**Published:** 2021-07-09

**Authors:** Yuanyue Li, Michael Kuhn, Joanna Zukowska-Kasprzyk, Marco L. Hennrich, Panagiotis L. Kastritis, Francis J. O’Reilly, Prasad Phapale, Martin Beck, Anne-Claude Gavin, Peer Bork

**Affiliations:** 1 Structural and Computational Biology Unit, European Molecular Biology Laboratory, Heidelberg, Germany; 2 Metabolomics Core Facility, European Molecular Biology Laboratory, Heidelberg, Germany; 3 Molecular Medicine Partnership Unit (MMPU), Heidelberg, Germany; 4 Max Delbrück Center for Molecular Medicine, Berlin, Germany; 5 Department of Bioinformatics, Biocenter, University of Würzburg, Würzburg, Germany; Aarhus University, DENMARK

## Abstract

Protein–metabolite interactions play an important role in the cell’s metabolism and many methods have been developed to screen them *in vitro*. However, few methods can be applied at a large scale and not alter biological state. Here we describe a proteometabolomic approach, using chromatography to generate cell fractions which are then analyzed with mass spectrometry for both protein and metabolite identification. Integrating the proteomic and metabolomic analyses makes it possible to identify protein-bound metabolites. Applying the concept to the thermophilic fungus *Chaetomium thermophilum*, we predict 461 likely protein-metabolite interactions, most of them novel. As a proof of principle, we experimentally validate a predicted interaction between the ribosome and isopentenyl adenine.

## Introduction

Interactions between proteins and endogenous metabolites are a hallmark of all cellular processes, from metabolism to signaling. In the former, enzymes interact with metabolites to catalyze chemical reactions, and in the latter, chemical compounds serve as co-factors for proteins to mediate protein function [1]. Protein–metabolite interactions have been historically discovered mostly individually, but more recently also by a variety of *in vitro* screening approaches [2,3]. Detection of interactions *in vivo* is much more difficult. Current methods mostly depend on overexpressing target proteins [4] or adding additional metabolite analogues, including thermal proteome profiling [5] and chemoproteomic approaches [6].

Several concepts have been developed to integrate proteomics and metabolomics on the same samples to find the relationship between proteins and metabolites [7,8]. Most methods focus on measuring free proteins and metabolites, then infer their associations, the associations can be indirect, unspecific and confounded. Few untargeted methods can be used directly to study protein–metabolite interactions *in vivo* without altering the biological state of the respective systems.

Recently, a method called PROMIS to detect endogenous protein–small molecule interactions *in vivo* has been presented and successfully applied in *Arabidopsis thaliana* [9]. In a similar vein, our method aims at the large-scaled, unbiased identification of direct and stable protein–metabolites interactions *in vivo*. We use size exclusion chromatography (SEC) to purify protein complexes [10] and their non-covalently bound metabolites in cell lysates. As a consequence, these fractions are free of unbound metabolites. Within the extracted fractions, proteins and metabolites are dissociated and separately identified using mass spectroscopy (MS)-based proteomics and metabolomics [11]. Improvements in *in silico* methods for metabolite identification allowed the assignment of many metabolites with high confidence [12,13]. Based on the correlations between paired elution profiles of proteins and metabolites we predict interactions between them.

## Results

**Fig 1:**
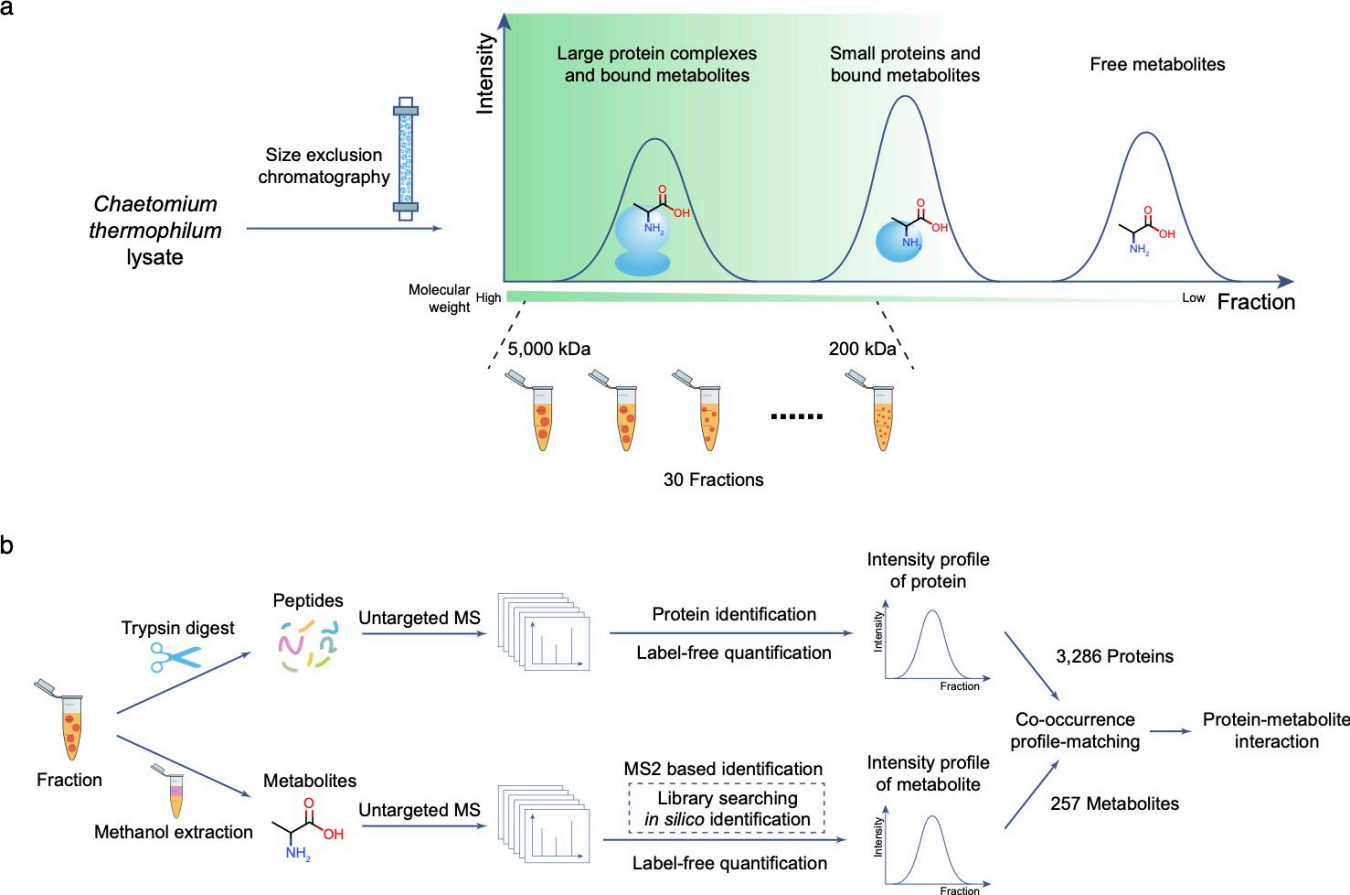
Workflow of the proteometabolomics experiment. (a) Cell lysate from *Chaetomium thermophilum* was separated by size exclusion chromatography and fractions with molecular weight between 200k Da and 5,000k Da were collected. (b) The collected fractions were divided into two parts, one part was digested by trypsin and analyzed by protein MS, the other part was extracted by methanol and analyzed by metabolite MS. Protein–metabolite interactions were inferred from the resulting intensity profiles.

We applied the concept to *Chaetomium thermophilum*, a thermophilic fungus and model organism for structural biology [14] as its protein complexes are particularly stable and is thus an ideal model organism for studying multimolecular interactions [15]. We grew *C*. *thermophilum* in standard medium, lysed the cells, and separated the crude native cell lysate with SEC. We then collected 30 size-fractions with molecular weights from 200 kDa to 5000 kDa (Fig 1A), which excludes small protein complexes and most individual proteins. As metabolites usually have a molecular weight below 1.5 kDa [16], those fractions can only contain metabolites which were bound to proteins or protein complexes. We split the collected fractions into two parts: one part was digested by trypsin and analyzed by bottom-up proteomics [17]. Altogether, 3,286 proteins were identified with high confidence (1% FDR), corresponding to 46% of the proteome. For the second part, chemical compounds were extracted by methanol and analyzed by untargeted mass spectrometry (Fig 1B). Tandem mass spectrometry (MS2) was used for compound identification: spectra were searched against public spectral databases for high-confidence identification. Furthermore, spectra were also searched by *in silico* identification methods [12,13] to maximize the rate of identification (S1 Fig). Altogether, we identified 257 metabolites in all fractions that were found to be bound to proteins or protein complexes (S1 Table). Prior to MS, metabolites were separated by hydrophilic interaction liquid chromatography (HILIC) according to their polarity. We were therefore able to compare the retention time in the HILIC column to the predicted polarity information (logP) to verify the accuracy of the compound identification (S2 Fig). We use known metabolite concentrations [18] in the well-studied *Saccharomyces cerevisiae* as a reference to estimate the identified metabolite concentration. This showed that our method can identify metabolites that have cellular concentrations higher than 100 μM (S3 Fig, see Methods).

**Fig 2:**
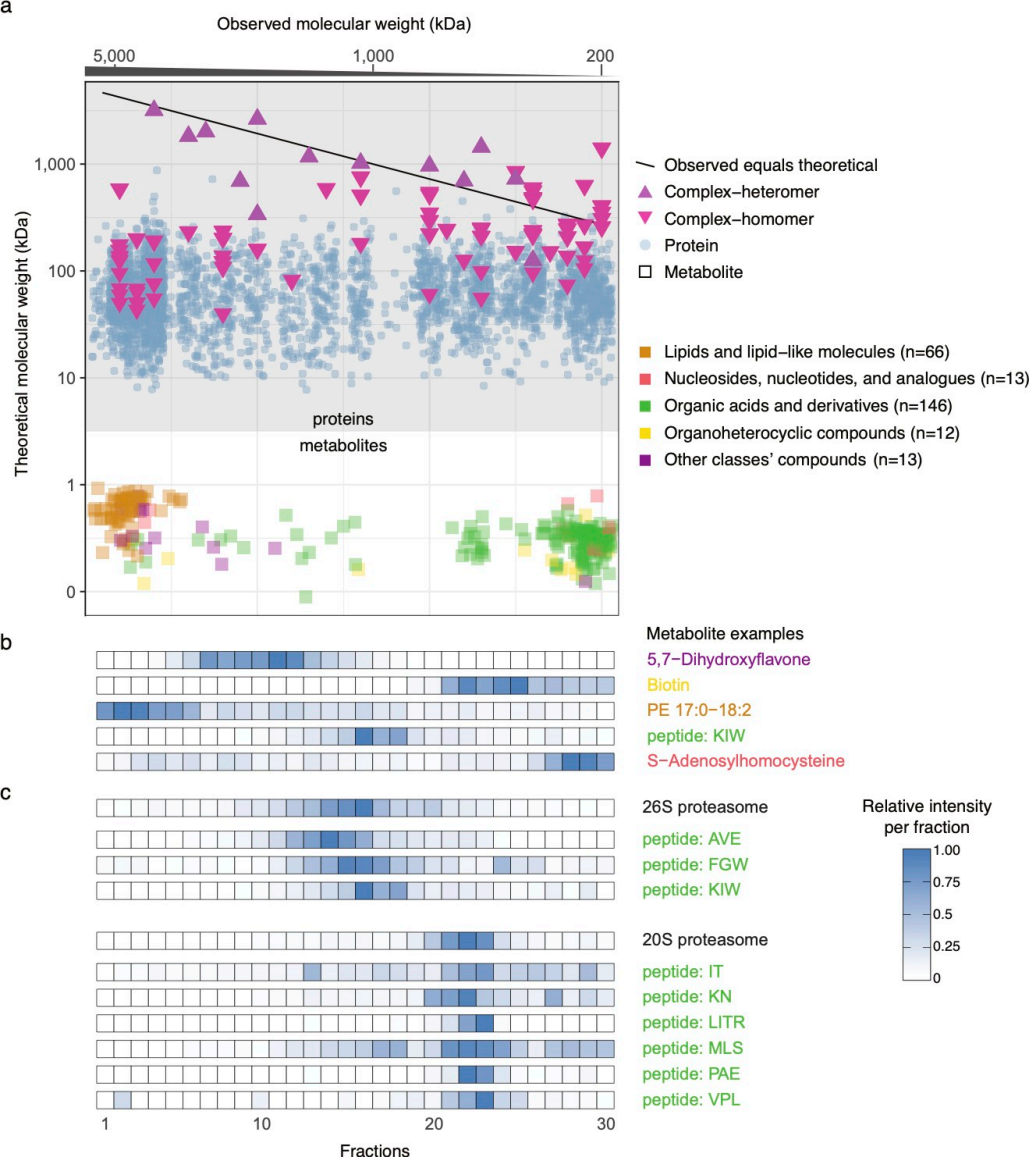
Identified proteins and metabolites. (a) For proteins (top part) and metabolites (bottom part), the relation between theoretical molecular weight and observed molecular weight (according to their elution time) is shown. When proteins are annotated with the molecular weight of heteromeric complexes that they participate in, there is a good correlation between the theoretical and observed molecular weights. Notably, metabolites are observed at molecular weights far above their actual molecular weight. (b) Relative intensity profiles for one example each from the five classes of identified metabolites. (c) Relative intensities of the 20S and 26S proteasomes and co-eluted peptides.

By comparing the theoretical molecular weights to the observed protein complex, we find a good agreement for heteromeric complexes, which suggest high quality of the data (Fig 2A). For individual proteins, homomeric complexes, and metabolites, the observed molecular weights are much higher than the theoretical molecular weight. This shows that proteins and compounds formed complexes of higher molecular weight.

We found that many lipids eluted in the high molecular weight fractions, which may be due to the formation of micelles during cell lysis [19]. We also identified many short peptides that could be the regulatory peptides [20] or the products of proteolysis (Fig 2B). With the exception of the lipids eluting in high molecular weight fractions, the metabolites were nearly equally distributed crossed different fractions in different molecular property (S4 Fig).

For every protein and metabolite, we determined their intensity in each fraction by label-free quantification. We found that the compounds have varying intensity profiles (Fig 2C). As the metabolites should be bound to proteins, a metabolite should have a similar elution profile as its protein binding partner. To pinpoint such interactions, we first identified protein communities using the method described by Kastritis *et al*. [15], which resulted in 95 protein communities. Then, we calculated the correlations of intensity profiles between all pairs of protein communities (or single proteins) and metabolites. By comparing protein–metabolite intensity profiles, we found that many peptides are associated with the 26S and 20S proteasomes (Fig 2C). During proteolysis, the proteasome generate fragments of lengths of two to ten amino acids [21], which we seem to capture. We verified that the observed peptides are likely to stem from the *in vivo* degradation of proteins rather than from the subsequent degradation of the proteasome itself during sample preparation by computing the relative frequencies of the tripeptides in the proteasome vs. the whole proteome (S5 Fig).

**Fig 3:**
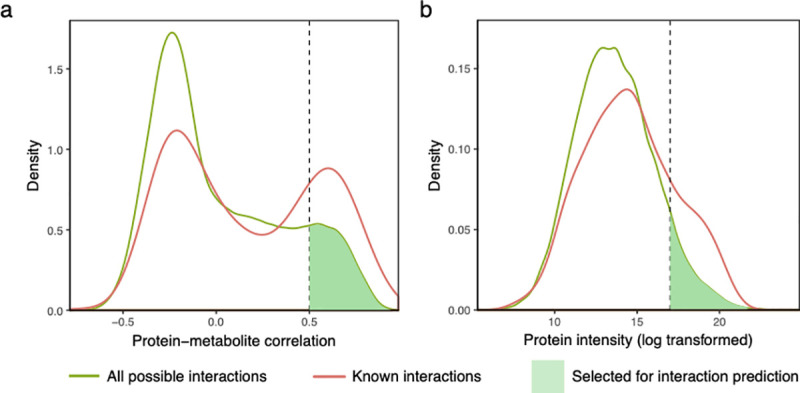
Distribution of correlations and intensities for protein–metabolite interactions. (a) The distribution of protein−metabolite correlations is bimodal, with known interactions showing increased correlations. We chose a cutoff of 0.5 for interaction predictions. (b) Proteins of higher intensity (i.e. abundance) are enriched among the known interactions. In both panels, the difference between the distributions is highly significant (p ≤ 2e-15 using Mann-Whitney *U* test).

Using 730 known protein–metabolite interaction data from the Brenda database [22], we observed an enrichment of known protein–metabolite interactions among highly correlated protein–metabolite pairs (p < 1-e15 with Mann-Whitney *U* test, Fig 3A). We further found that highly abundant proteins are more likely to have known interactions in the Brenda database for which we can detect the binding partner (p = 2e-15 with Mann-Whitney *U* test, Fig 3B). This may be partially due to the bias in existing data towards more abundant proteins, but also due to the experimental design that makes it more likely to detect proteins and compounds in sufficient amounts and fidelity when the protein is more abundant.

**Fig 4:**
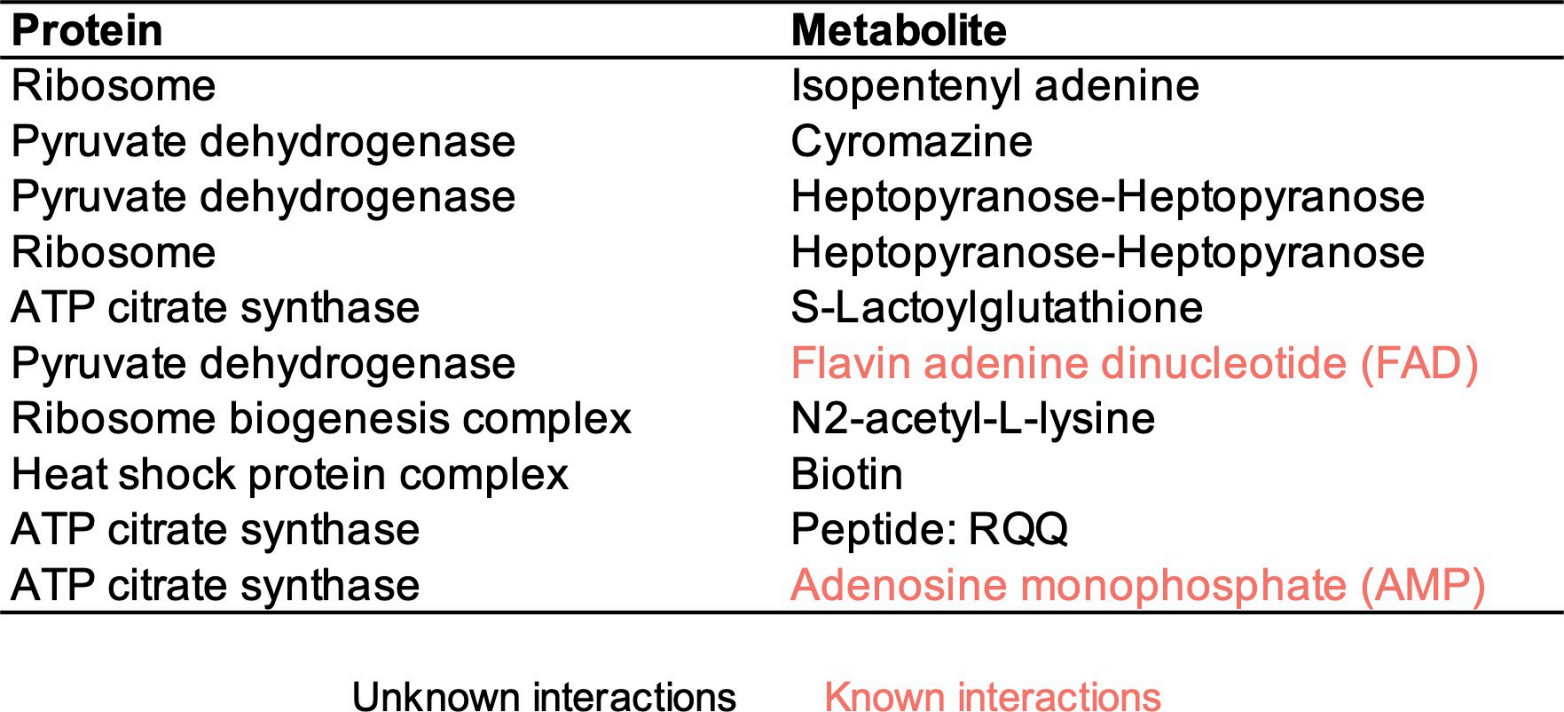
The top 10 scoring protein–metabolite interactions. Known interactions are shown in red, and proposed novel interactions are shown in black. One of the identified compounds, namely cyromazine, is an insecticide. It is not clear whether this compound has been introduced as part of the growth medium, or if this is a mis-annotation of an endogenous compound.

**Fig 5:**
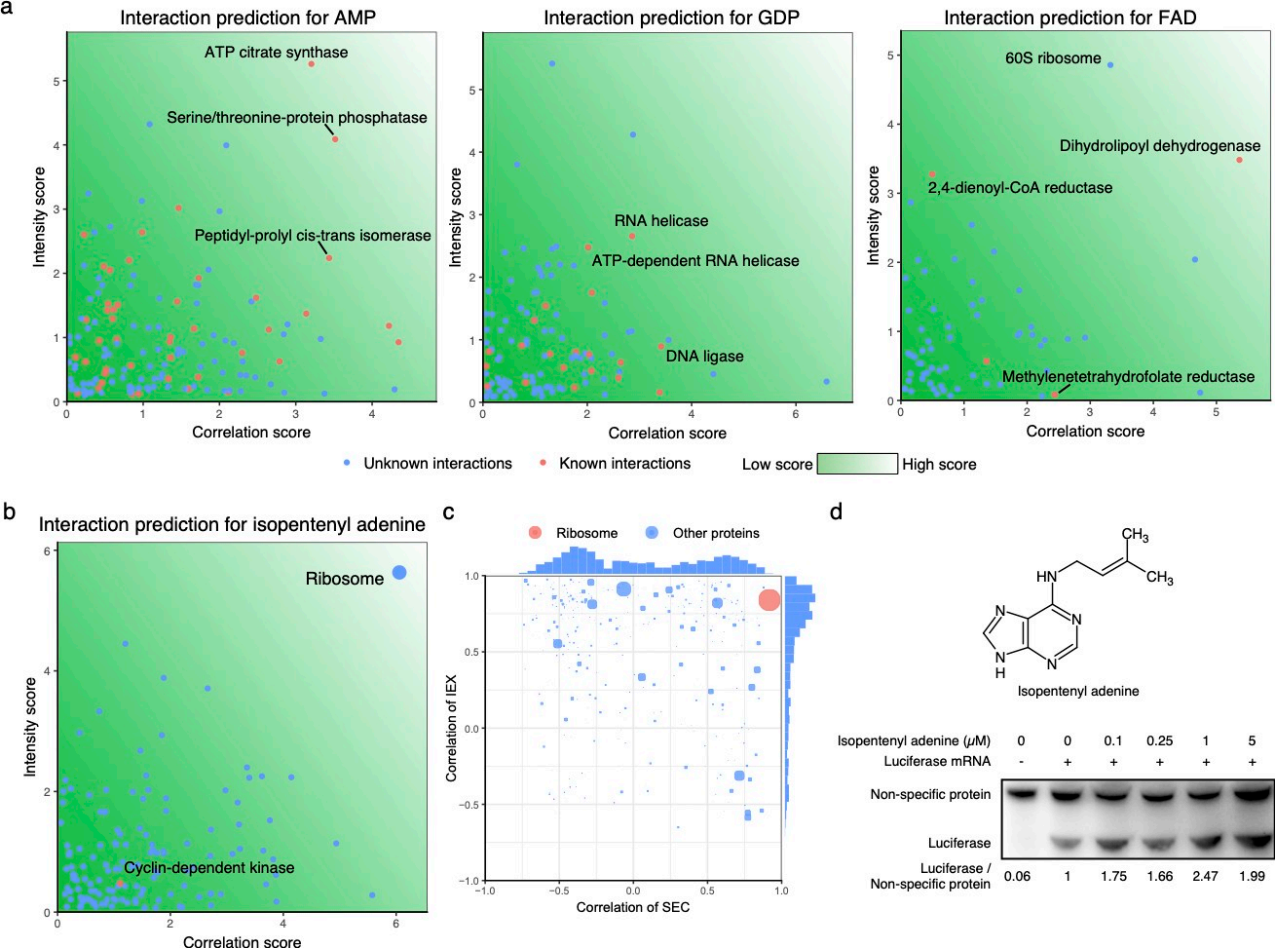
The predicted protein–metabolite interactions. (a) For three example proteins, predicted and known interactions with metabolites are shown. (b) The intensity score and correlation of all possible proteins which can interact with the metabolite isopentenyl adenine. Among all those proteins, the ribosome has the highest score. (c) Correlations between candidate proteins and isopentyl adenine are shown after independently performing IEX and SEC. Among all proteins, the ribosome has the highest score. (d) The experimental verification of the interactions between the ribosome and isopentenyl adenine: In the *in vitro* transcription system, luciferase was used as reported protein. Ribosome activity was measured by calculating the ratio of luciferase/non-specific protein.

For protein–metabolite interaction prediction, we removed the data from the last three fractions. Too many proteins co-eluted in these fractions, making it difficult to predict protein–metabolite interactions with high confidence. As microsomes and micelles eluted in the early fractions, we removed lipids from these to avoid a false signal from lipids contained in the microsomes. We then calculated two scores for each candidate interactions: a correlation score based on the Pearson correlation between the protein and metabolite intensity profiles; and an intensity score based on the protein’s abundance. For each identified metabolite, we calculated these two scores and their respective empirical distribution function of the correlation and intensity. We used Fisher’s linear discriminant to find the best combination of the correlation score and intensity score as benchmarked with known protein–metabolite interactions and selected the top 10% as high confidence predictions (Fig 4, S2 Table). For example, FAD is known to interact with dihydrolipoyl dehydrogenase as a cofactor in the pyruvate dehydrogenase complex [23] (Fig 5A). Some metabolites have many interaction partners, for example AMP. In these cases, not all interaction partners can be highly correlated, but our method will pick up the proteins with the highest amount of bound metabolite (due to a combination of protein concentration and binding affinity). Therefore, our method is most suited to propose interaction partners for metabolites that are binding to a small number of proteins.

To validate our predicted protein–metabolite interactions, we focused on the interaction between isopentenyl adenine and ribosome as it has both very high correlation score and intensity abundance score (Fig 5B). First, a two-step chromatography experiment was preformed: we used ion-exchange chromatography (IEX) to separate the cell lysate. Then, all fractions were separated by SEC. We found that isopentenyl adenine still co-eluted with the ribosome (Fig 5C) in the two-step chromatography experiment. We further measured the ribosome’s activity by an *in vitro* transcription assay in a wheat germ extract system (Figs 5D and S6), as the system is well established. We found that ribosomal activity increased when isopentenyl adenine is present, supporting the predicted interaction. In one experiment, one data point could not be used due to a failure in the Western blot, but it is clear that the ribosome activity was increased. In all three other experiments, we could confirm a statistically significant increase using one-tailed one-sample *t*-test (S6 Fig).

## Discussion

We present a proof of concept that the combination of SEC, untargeted proteomic, and metabolic MS is able to identify physical *in vivo* protein–metabolite interactions. Our integrated approach does not rely on modifications to either proteins or metabolites and investigates cell in its native state. It can therefore be easily adapted to other organisms, both uni- and multicellular. Compared to the PROMIS method, our approach independently affirms the feasibility of the concept, and goes beyond it by adding a combined ranking of candidates based on protein–metabolite correlations and protein abundances. There are several possibilities to improve the method. To refine the scoring system, to discover additional interactions, and to study the impact of environmental changes, a next step would be to subject cells to a variety of conditions such as changes in medium, temperature, oxygen content etc. The resolution of the method can be further increased by adding further fractionation steps. Nevertheless, the concept presented here is already an entry point for large-scale detection of endogenous metabolites bound to proteins or their complexes.

## Methods

### Cell growth, lysis and size exclusion chromatography

Cells were grown as previously described by Kastritis et al. [15]. *Chaetomium thermophilum* was obtained from Deutsche Sammlung von Mikroorganismen und Zellkulturen (DMSZ No.: 1495). In brief, 2 l *Chaetomium thermophilum* var. *thermophilum* were grown in LB medium, 50°C and 10% CO_2_. 25 g cells were collected and lysed by freeze-grinding in liquid nitrogen in lysis buffer (100 mM HEPES pH 7.4, 95 mM NaCl, 5 mM KCl, 5% glycerol, 1 mM MgCl_2_, 0.5 mM EDTA, 1 mM DTT, 10 μg/ml DNAse, pefabloc 2 mM, E-64 2 μM, Bestatin 10 μM, Aprotinin 0.3 μM, Leupeptin 1 μM, pepstatin A 1.45 μM). The lysate was centrifuged at 100,000g for 45min to remove cell debris and concentrated with a 100 kDa Amicon Ultra centrifugal filter. 0.5 ml concentrated lysate (approximately 30 mg/ml) was separated by a Biosep SEC-S4000 (7.8 mm x 600 mm) size exclusion column in 100 mM HEPES pH 7.4, 95 mM NaCl, 5 mM KCl, 1 mM MgCl_2_. The fractions were collected from 10.5mL to 18mL, with 0.25 ml per fraction. For every sample, the cell lysate was separated in three separate SEC runs. The corresponding fractions were pooled to get a final volume of 0.75 ml per fraction.

### Untargeted protein identification by mass spectrometry

40 μl of each fraction were subjected to a tryptic in-solution digest as previously described [24,25]. 2 μl of 20% SDS were added to avoid precipitation of proteins during the reduction and alkylation of proteins. Proteins were reduced by the addition of 1 μl of 200 mM DTT in 200 mM Hepes/NaOH pH 8.5 following incubation for 30 min at 56°C. Subsequently, 2 μl of 400 mM chloroacetamide in 200 mM Hepes/NaOH, pH 8.5 were added and samples were incubated for 30 min at 25°C before excess chloroacetamide was quenched by the addition of 2 μl of 200 mM DTT in Hepes/NaOH, pH 8.5. For the in-solution digest, the reduced and alkylated samples were subjected to the Single-Pot Solid-Phase-enhanced Sample Preparation (SP3) protocol [24,25]. To this end, 2 μl of Sera-Mag Beads, and 5 μl of 10% formic acid (v/v) were added. Acetonitrile (ACN) was added to achieve a final ACN percentage of 50%. Samples were incubated for 8 min before beads were captured on a magnetic rack. Beads were washed twice with 200 μl 70% ethanol and once with 200 μl ACN. Beads were resuspended in 10 μl of 0.8 μg of sequencing grade modified trypsin in 10 μl 100 mM Hepes/NaOH, pH 8.5 following overnight incubation at 37°C. Peptides were subjected to a reverse phase clean-up step and analyzed by LC-MS/MS on a Q Exactive Plus.

Samples were analyzed with liquid chromatography coupled to tandem mass spectrometry. Peptides were separated using an UltiMate 3000 RSLC nano-LC system equipped with a trapping cartridge and an analytical column. Solvent A was 0.1% formic acid in LC-MS grade water and solvent B was 0.1% formic acid in LC-MS grade acetonitrile. After loading the peptides onto the trapping cartridge (30 μl/min of solvent A for 3 min), elution was performed with a constant flow of 0.3 μL/min using 90 min analysis time (with a 2–28% B elution, followed by an increase to 40% B,80% B washing step and re-equilibration to initial conditions). The LC system was directly coupled to a Q Exactive Plus mass spectrometer using a Nanospray-Flex ion source and a Pico-Tip Emitter 360 μm OD x 20 μm ID; 10 μm tip. The mass spectrometer was operated in positive ion mode with a spray voltage of 2.3 kV and a capillary temperature of 275°C. Full scan MS spectra with a mass range of 350–1400 m/z were acquired in profile mode using a resolution of 70,000 [maximum fill time of 100 ms or a maximum of 3e6 ions (automatic gain control, AGC)]. Fragmentation was triggered for the top 20 peaks with charge 2 to 4 on the MS scan (data-dependent acquisition) with a 20 s dynamic exclusion window (normalized collision energy was 26). Precursors were isolated with 1.7 m/z and MS/MS spectra were acquired in profile mode with a resolution of 17,500 (maximum fill time of 50 ms or an AGC target of 1e5 ions). For the data analysis, the MS raw data were analyzed by MaxQuant 1.6.1 [26]. *Chaetomium thermophilum* proteomes sequences were downloaded from Uniprot with Proteome ID UP000008066. The MS data were searched against *Chaetomium thermophilum* proteomes sequences plus common contaminants sequence provided by MaxQuant. The default setting of MaxQuant was used with modification oxidation and acetyl (protein N-term). A false-discovery rate (FDR) cutoff of 1% was used for protein identification, and iBAQ intensity was used for label-free protein quantitation. When calculating iBAQ intensity, the maximum detector peak intensities of the peptide elution profile were used as the peptide intensity. Then, all identified peptide intensities were added and normalized by the total number of identified peptides.

### Metabolite extraction and untargeted mass spectrometry

10 μl 250 ppm ^13^C-creatinine was added into 650 μl fractions as spike-in control. Then, methanol was added up to a final concentration to 80%. The sample was centrifugated at 14,000 g for 20 min, then the supernatant was collected and concentrated with a speed vacuum concentrator to get 200 μl final volume.

LC-MS/MS analysis was performed on a Vanquish UHPLC system coupled to a Q-Exactive plus HRMS in both ESI positive and negative mode. The separation of metabolites was carried out on Xbridge Amide (100 X 2.1 mm; 2.6 uM) at a flow rate of 0.3 ml/min and maintained at 40°C. The mobile phase consisted of solvent A (7.5 mM Ammonium acetate with 0.05% NH_4_OH) and solvent B (acetonitrile). The UHPLC system was run in gradient mode as follows: 0 min, 85% B; 2 min, 85% B; 12 min, 10% B; 14 min, 10% B; 14.1 min 85% B; 16min 85% B.

Metabolites were detected with HRMS full scan at the mass resolving power R = 70000 in the mass range of 60–900 m/z. The data-dependent tandem (MS/MS) mass scans were obtained along with full scans using higher energy collisional dissociation (HCD) of normalized collision energies of 10, 20 and 40 units which were at the mass resolving power R = 17500. The MS parameters in the Tune software were set as follows: spray voltage of 4 kV (for negative mode 3.5 kV), sheath gas 30 and auxiliary gas 5 units, S-Lens 65 eV, capillary temperature 320°C and vaporization temperature of auxiliary gas was 300°C. Data was acquired in full scan mode and data dependent tandem mass spectra (MS/MS) for top 10 most intense precursors ions.

### Data analysis for untargeted metabolite mass spectrometry

The MS raw file was converted to mzML file by MSConvert [27] and MS features were extracted from mzML files by XCMS [28]. The charge of the MS features was determined by comparing the isotopic peaks; features with charge > 1 were discarded. The feature’s intensities across different runs were normalized by spike-in intensities and smoothed across different fractions by the median filter (window size: three fractions).

A feature is considered only if it is found in both replicates. Furthermore, we required intensity profiles of the feature across all the fractions to have a Pearson correlation greater than 0.5 between the two replicates. As a final filtering step, we determined the signal-to-noise ratio of features as follows: the maximum value of the smoothened intensity profile was considered as “signal.” We applied another smoothing step (median filter, window size: six fractions) and took the minimum value as “noise”. Metabolites with signal-to-noise ratio above five were selected.

### Metabolite identification for the metabolite mass spectrometry

Metabolite MS/MS spectra were searched against public databases (GNPS [29], Metlin [30], MassBank [31]) and an internal spectral database [32]. The weighted matching score was used to calculate the match between reference spectrum and experimental spectrum, using an FDR cutoff of 10% was for metabolite identification.

Sirius 4 [13] and SF-Matching [12] were used for *in silico* identification: spectra corresponding to the features selected above were searched against a combined database containing all molecules from KEGG, HMDB, ChEBI, and ChEMBL, plus all possible dipeptides, tripeptides, and tetrapeptides. To improve identification accuracy, an identified metabolite was considered as valid only when it was the consensus result of Sirius 4 and SF-Matching.

### Metabolite analysis

For the metabolite classification, we converted the molecular structure into InChI key and used the ClassyFire website (http://classyfire.wishartlab.com) [33] to assign the metabolites to classes. The metabolite’s LogP was calculated by the Crippen approach in rdkit packages [34]. The approximate concentration of metabolites was retrieved from the Yeast Metabolome Database (YMDB) [18]. We excluded the condition “YEB media with 0.5 mM glucose” as its distribution of concentrations differed from all other reported conditions.

### Protein–metabolite interaction prediction

As last three fractions contain many protein and metabolites, to get high confidence protein-metabolite interactions, we use the data from the first 27 fractions. We calculated two separate scores for protein–metabolite pairs, based on their correlation and based on the protein’s abundance. For the correlation score, first, the intensities of protein and metabolite across all fractions were calculated and smoothed between fractions by a median filter (window size: 3 fractions). Then, Pearson correlations between all identified proteins and metabolites were calculated and protein–metabolite pairs with correlation greater than 0.5 were selected. From correlations of these 10,251 protein–metabolite pairs, we derived the empirical distribution function (EDF). The correlation score of a single protein–metabolite pair is the value of the EDF at the pair’s correlation. To calculate the abundance score for a given protein–metabolite pair, we determined the fraction which had the highest intensity of this compound. Then, the protein intensity in this fraction was calculated, and the empirical distribution function was determined based on the intensities of all proteins across all fractions.

In order to calculate a weighted combination of the two scores, for all possible protein-metabolite pairs, we log-transformed both scores (using the natural logarithm). Then, all the known protein-metabolite interactions from the Brenda database [22] were treated as the positive interactions. The Fisher’s linear discriminant analysis was preformed to find a combination of weights to maximize the distances between the positive and negative interactions. The final protein-metabolite interaction scores were calculated by the weights, and the top 10% scores were selected as the high confidence predictions. As first few fractions contain lipid from micelles during cell lysis, we removed the interactions containing lipids which mainly eluted in the first five fractions, which results in 461 protein-metabolites interactions.

### Two-step chromatography

The *Chaetomium* cell lysate was separated by ion-exchange chromatography first. A 5 mL HiTrap Q XL column (GE) was first equilibrated with buffer A (25 mM pH 7.4 Hepes, 23.75 mM NaCl, 2.5% Glycerol, 2.5 mM KCl, 0.5 mM MgCl_2_). After injection of the sample, the column was washed with the buffer A. Then, bound proteins were eluted with buffer A containing a NaCl gradient (from 25 mM Cl^-^ to 1 M Cl^-^). Four fractions were collected in total. The second chromatographic step was performed using the size exclusion chromatography described above. To enrich for isopentenyl adenine, we collected only the first ten fractions were collected, which contain most of the compound. These ten SEC fractions were pooled and analyzed with proteomics and metabolomics as described above.

### *In vitro* translation assay

Isopentenyl adenine (Sigma) and Transcend™ biotinylated lysine tRNA (Promega) were added into the *in vitro* translation wheat germ systems (Promega). The mixed reaction system was incubated at 27°C for 1 or 2 hours. The synthesized proteins were biotinylated, and then separated and detected by Western blot. The HRP-conjugated streptavidin (Sigma) chemiluminescent detection system was used to visualize the biotinylated proteins.

## Supporting information

S1 Fig Venn diagram of the number of identified metabolites from different methods. (PDF)

S2 Fig Comparison of theoretical and observed molecular polarity. (PDF)

S3 Fig Distribution of metabolite concentrations in S. cerevisiae. The red line shows the distribution all metabolites in the Yeast Metabolome Database. Blue bars show the concentration of metabolites that we could identify in our experiments.
(PDF)

S4 Fig The identified metabolites across different fractions. (a) The m/z profile of identified metabolites. (b) The retention time profile of identified metabolites. (c) The LogP profile of identified metabolites.
(PDF)

S5 Fig Probability of tripeptides originating from the proteasome itself. All possible tripeptides were searched against the whole proteome to compute the probability of the peptide originating from proteasome. If the identified tripeptides were the result of digestion or degradation of the proteasome itself, then we would expect them to be enriched among higher probabilities. This, however, was not the case.
(PDF)

S6 Fig Isopentenyl adenine can increase the ribosome’s activity *in vitro*. (a) Western blot of four replicates experiments. (b) Quantification of western blot results. The intensity of each band was determined by Image Lab Software from Bio-Rad. The relative ribosomal activity is calculated by dividing the intensity of luciferase band to the intensity of the non-specific band, then normalized by the control which does not contain Isopentenyl adenine. A point from experiment 1, 1 μM isopentenyl adenine, is removed due to the failure of Western blot experiment. For experiments 2 to 4 (which have at least three data points), we evaluated whether there is a significant increase in ribosome activity using a one-tailed one-sample *t*-test between the treatment conditions and the untreated control. This resulted in p-values of 0.0006, 0.072, and 0.0163, respectively (raw data in S3 Table). A clearly monotonic dose response could be observed in all three experiments with 60 minutes incubation time.
(PDF)

S1 Table Identified metabolites. For each metabolite, its name, precursor ion m/z, retention time, charge, metabolite class, InChI key and InChI are listed.
(XLSX)

S2 Table Proposed protein–chemical interactions. For all proposed interactions, the table contains: Metabolite (identified by name, InChI key, and InChI), protein community (using protein identifiers), interaction score, and whether the interaction is known according to the Brenda database.
(XLSX)

S3 Table Western blot quantification. This table contains the raw intensity measurements for S6 Fig.
(XLSX)

S1 Raw images Raw images for Western blots. This file contains the raw images for S6 Fig.
(PDF)
