# Supplementary material for: Coupling proteomics and metabolomics for the unsupervised identification of protein–metabolite interactions in Chaetomium thermophilum
Source: PLoS One. 2021 Jul 9;16(7):e0254429. doi: 10.1371/journal.pone.0254429 (PMC8270407; doi:10.1371/journal.pone.0254429)
Supplement: S1 Fig — (PDF) [file pone.0254429.s001.pdf]

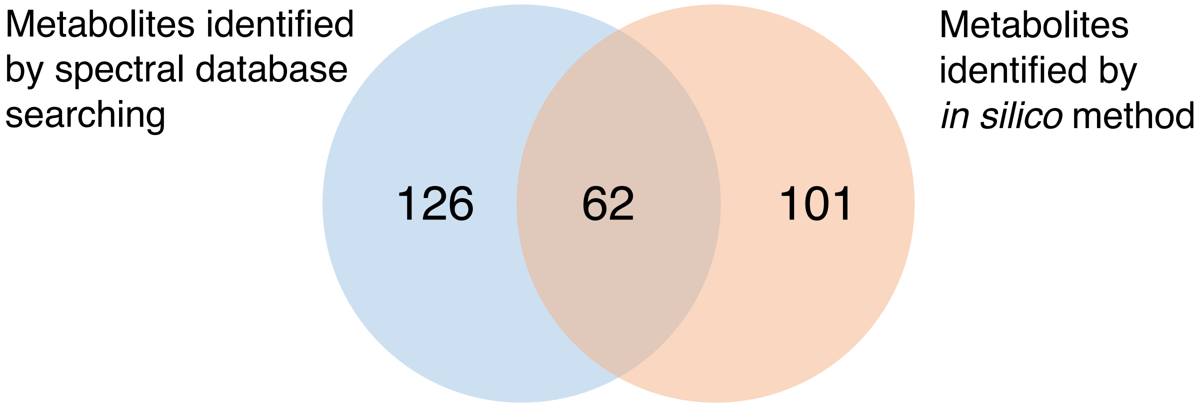
Metabolites identified by spectral database searching
126
62
101
Metabolites identified by *in silico* method
