# Supplementary figures and images for: Coupling proteomics and metabolomics for the unsupervised identification of protein–metabolite interactions in Chaetomium thermophilum
Source: PLoS One. 2021 Jul 9;16(7):e0254429. doi: 10.1371/journal.pone.0254429 (PMC8270407; doi:10.1371/journal.pone.0254429)

# Identified metabolites

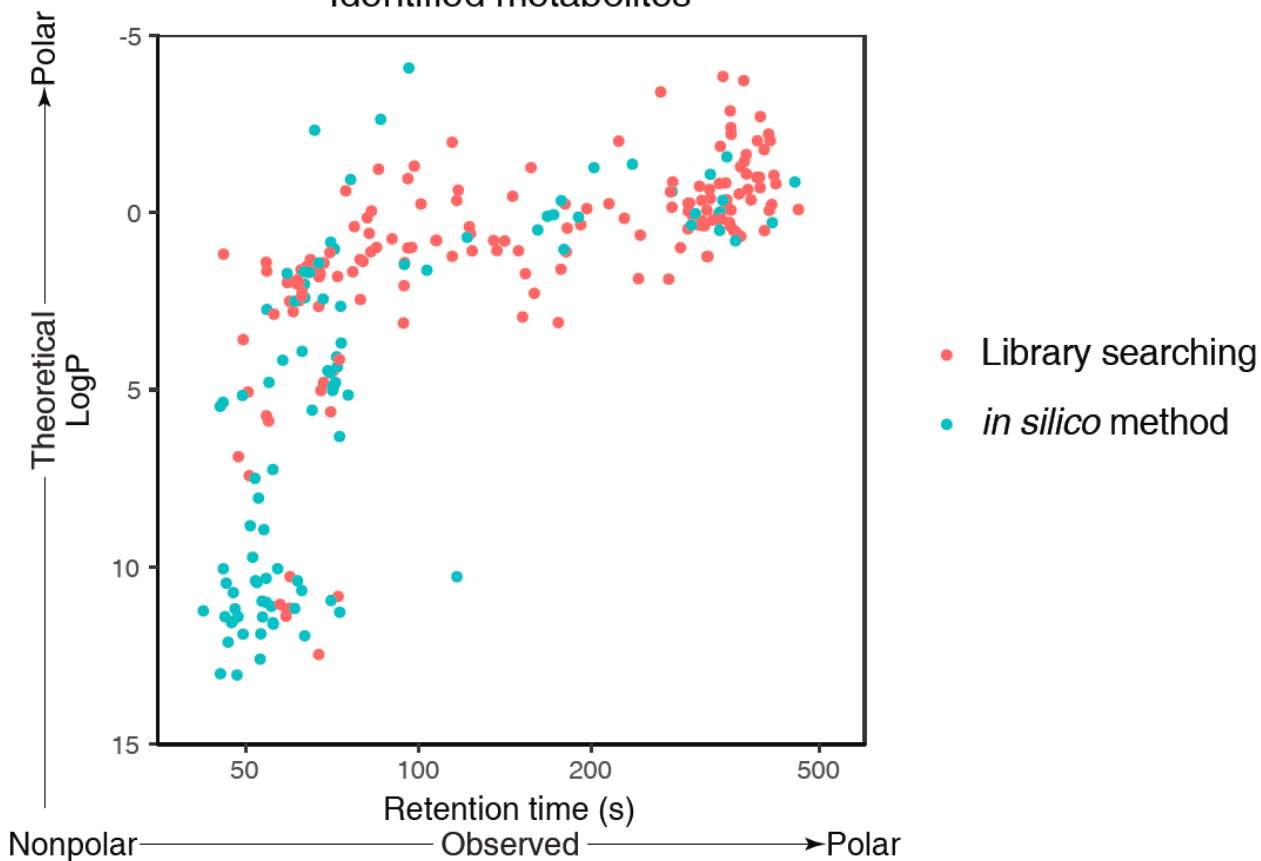

Supplement: S2 Fig — (PDF) [file pone.0254429.s002.pdf]

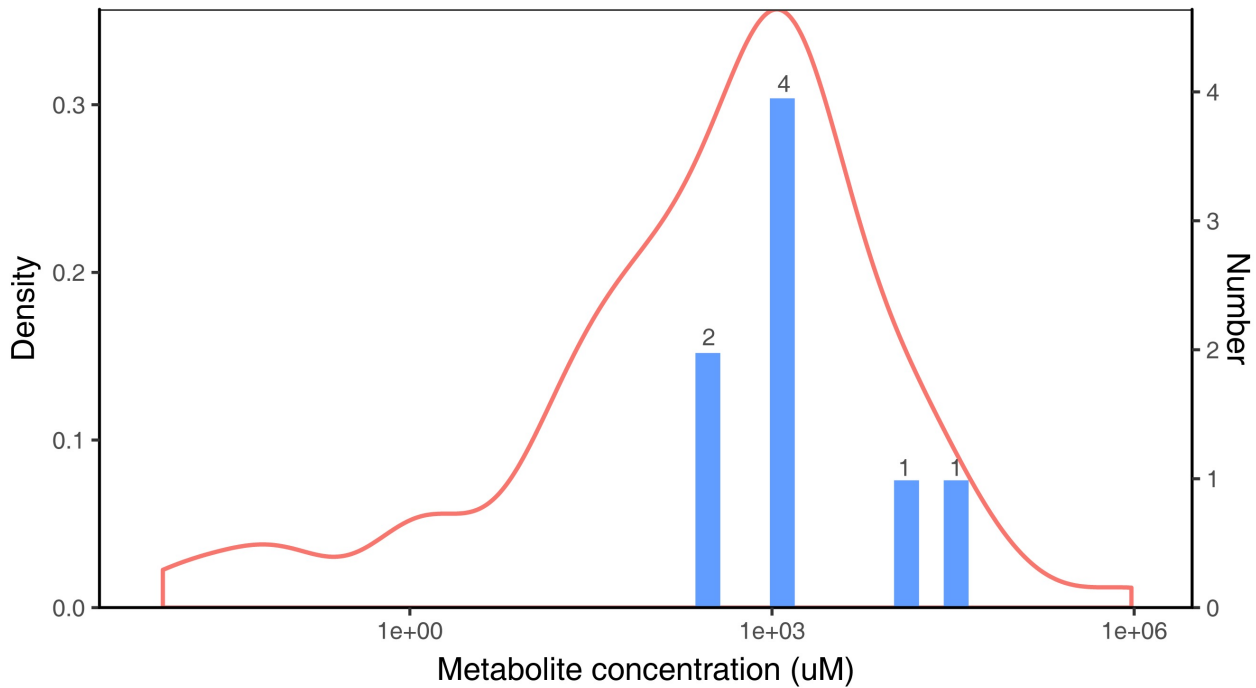

Supplement: S3 Fig — The red line shows the distribution all metabolites in the Yeast Metabolome Database. Blue bars show the concentration of metabolites that we could identify in our experiments. (PDF) [file pone.0254429.s003.pdf]

Probability of coming from proteasome

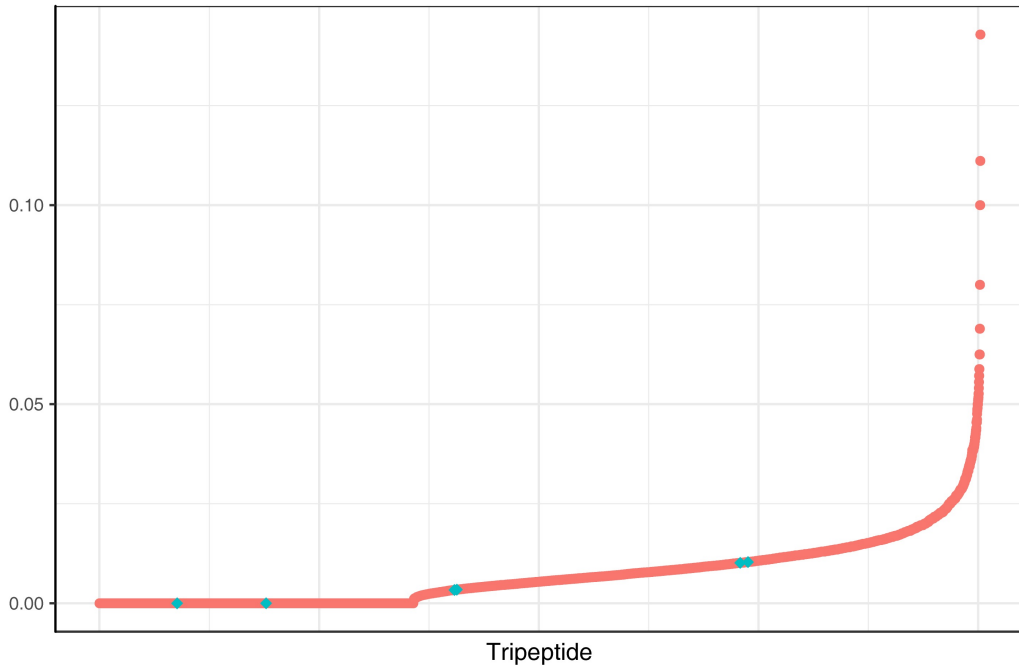

Supplement: S5 Fig — All possible tripeptides were searched against the whole proteome to compute the probability of the peptide originating from proteasome. If the identified tripeptides were the result of digestion or degradation of the proteasome itself, then we would expect them to be enriched among higher probabilities. This, however, was not the case. (PDF) [file pone.0254429.s005.pdf]

a

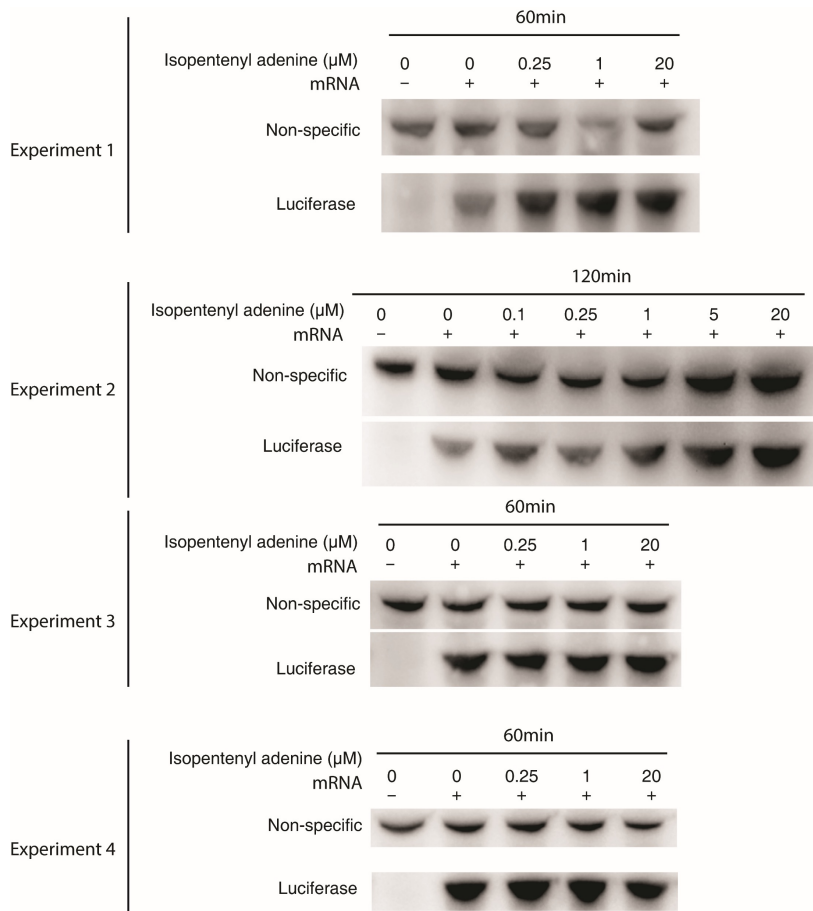

b

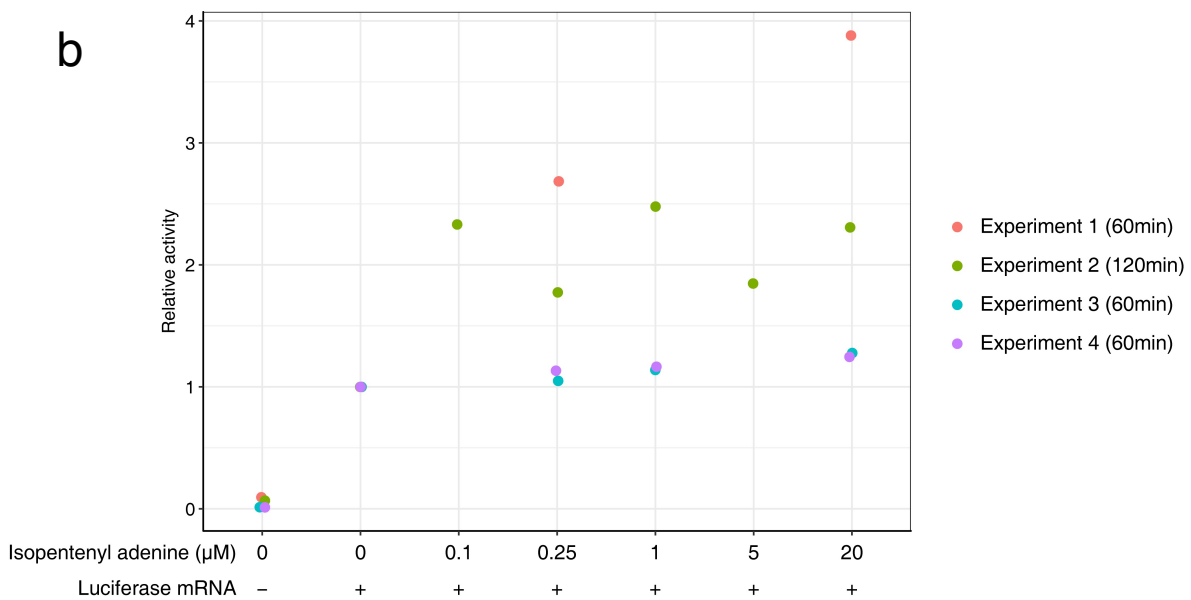

Supplement: S6 Fig — (a) Western blot of four replicates experiments. (b) Quantification of western blot results. The intensity of each band was determined by Image Lab Software from Bio-Rad. The relative ribosomal activity is calculated by dividing the intensity of luciferase band to the intensity of the non-specific band, then normalized by the control which does not contain Isopentenyl adenine. A point from experiment 1, 1 μM isopentenyl adenine, is removed due to the failure of Western blot experiment. For experiments 2 to 4 (which have at least three data points), we evaluated whether there is a significant increase in ribosome activity using a one-tailed one-sample t-test between the treatment conditions and the untreated control. This resulted in p-values of 0.0006, 0.072, and 0.0163, respectively (raw data in S3 Table). A clearly monotonic dose response could be observed in all three experiments with 60 minutes incubation time. (PDF) [file pone.0254429.s006.pdf]

# Experiment 1

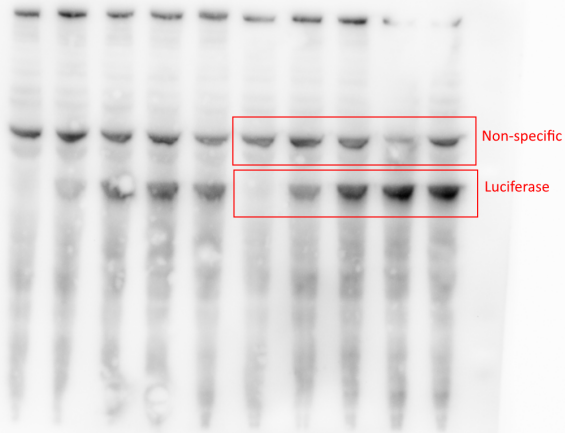

## Experiment 2

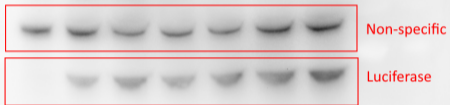

## Experiment 3

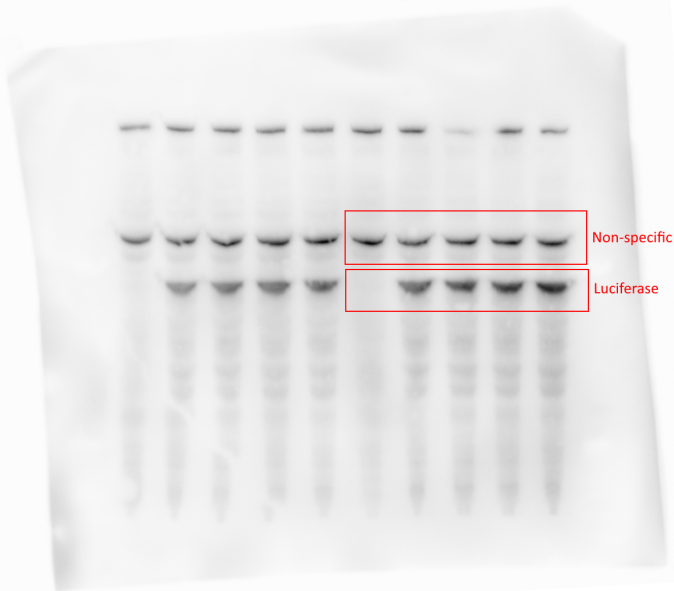

# Experiment 4

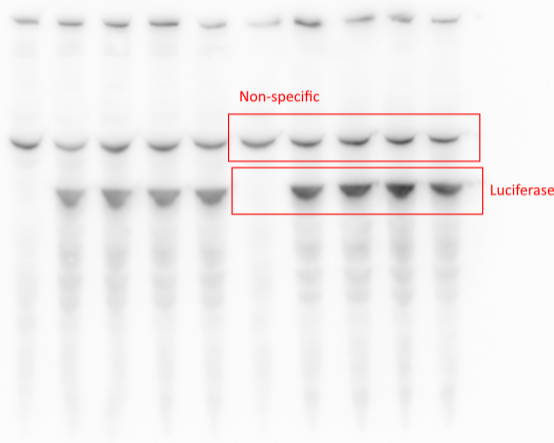

Supplement: S1 Raw images — This file contains the raw images for S6 Fig. (PDF) [file pone.0254429.s010.pdf]
