# Supplementary material for: Coupling proteomics and metabolomics for the unsupervised identification of protein–metabolite interactions in Chaetomium thermophilum
Source: PLoS One. 2021 Jul 9;16(7):e0254429. doi: 10.1371/journal.pone.0254429 (PMC8270407; doi:10.1371/journal.pone.0254429)
Supplement: S4 Fig — (a) The m/z profile of identified metabolites. (b) The retention time profile of identified metabolites. (c) The LogP profile of identified metabolites. (PDF) [file pone.0254429.s004.pdf]

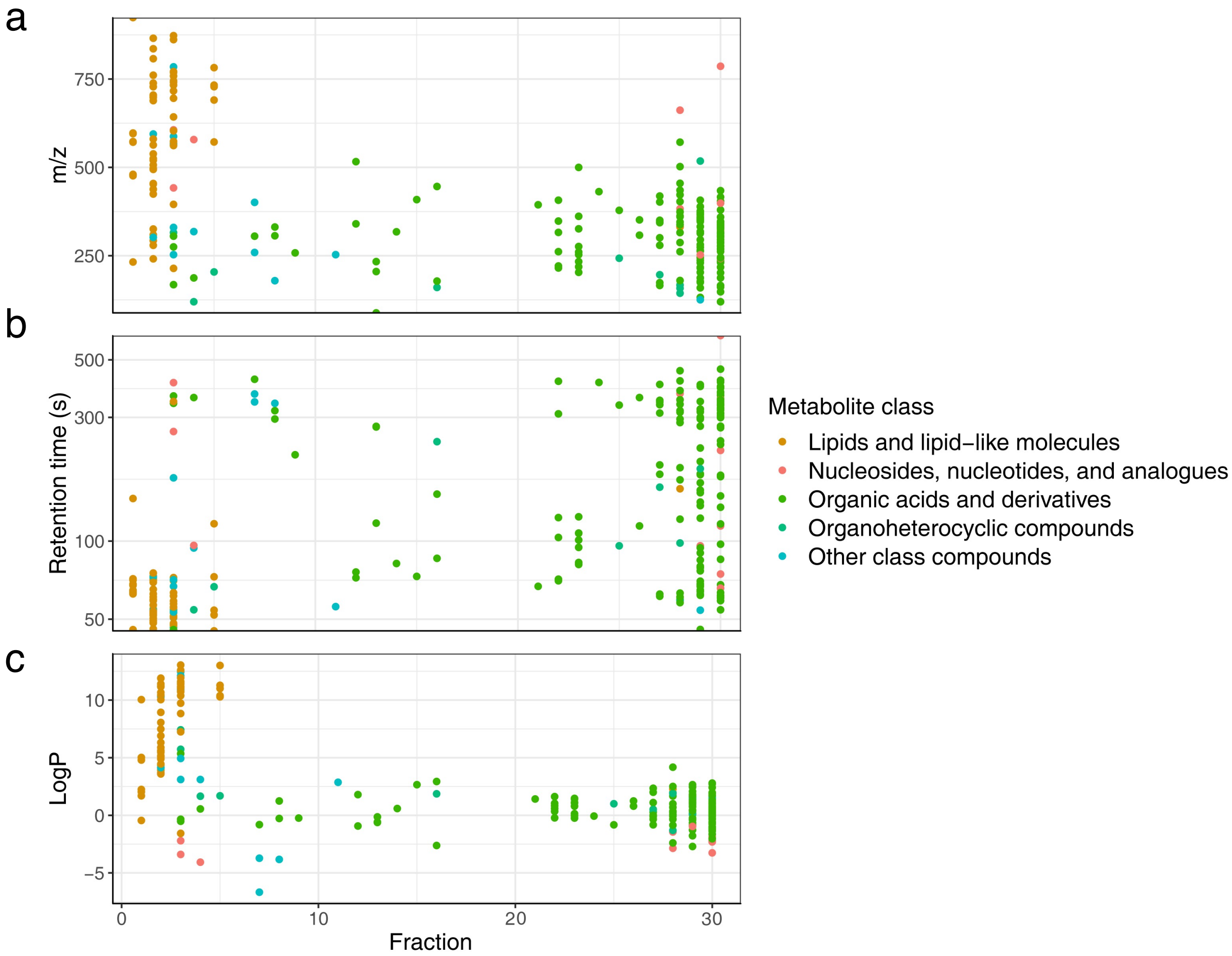
a
b
c
m/z
750
500
250
Retention time (s)
500
300
100
50
LogP
10
5
0
−5
0
10
20
30
Fraction
Metabolite class
Lipids and lipid−like molecules
Nucleosides, nucleotides, and analogues
Organic acids and derivatives
Organoheterocyclic compounds
Other class compounds
